# Pharmacokinetics and Protective Effects of Tartary Buckwheat Flour Extracts against Ethanol-Induced Liver Injury in Rats

**DOI:** 10.3390/antiox9100913

**Published:** 2020-09-24

**Authors:** Hye-Rin Jin, Suyong Lee, Soo-Jin Choi

**Affiliations:** 1 Division of Applied Food System, Major of Food Science & Technology, Seoul Women’s University, Seoul 01797, Korea; ckatkfkd09@swu.ac.kr; 2 Department of Food Science and Biotechnology, Sejong University, Seoul 05006, Korea; suyonglee@sejong.ac.kr

**Keywords:** Tatary buckwheat flour extract, in vivo antioxidant activity, alcoholic liver disease, protective effect

## Abstract

The grains of Tartary buckwheat (*Fagopyrum esculentum*) are traditionally consumed on a daily basis and are used in the preparation of diverse processed foods owing to the high concentration of rutin, an antioxidant compound. However, rutin is highly concentrated in hull and bran, but not in edible flour fractions. Rutin-enriched TB flour extracts (TBFEs) were obtained by hydrothermal treatment (autoclaving, boiling, or steaming) and their pharmacokinetic profiles were evaluated following a single-dose oral administration in rats. The antioxidant and protective activities of the extracts against alcoholic liver disease (ALD) were investigated after repetitive oral administration of TBFEs for 28 days prior to ethanol ingestion. The results demonstrated that rutin-enriched TBFEs had better oral absorption and was retained longer in the bloodstream than native TBFE or standard rutin. The activities of antioxidant enzymes and intracellular antioxidant levels increased in ALD rats following TBFE treatments, especially following the administration of rutin-enriched TBFEs. The antioxidant activity of TBFEs consequently contributed toward protecting the liver against injury caused by repetitive ethanol administration, as confirmed by analyzing relative liver weight, liver injury markers, lipid peroxidation, and calcium permeability. These results suggest the promising potential of TBFEs as antioxidant-enriched functional foods for human health.

## 1. Introduction

Tartary buckwheat (TB, *Fagopyrum esculentum*) is grown in southern China, India, South Korea, Nepal, Bhutan, Pakistan, etc. Traditionally, the grains of TB are extensively consumed on a daily basis in the form of noodles, gels, and breads [1,2]. This is owing to the fact that they are rich in proteins, vitamins, minerals, dietary fibers, and functional components such as rutin, quercetin, and other flavonoids [3,4]. In particular, TB has high rutin content compared with common buckwheat [3,5]. Rutin (quercetin-3-0-rutinoside) is a glycoside form comprising quercetin and disaccharide rutinose. Several reports have demonstrated that rutin helps blood circulation, prevents blood clots, lowers cholesterol, and reduces arthritic pain and these benefits are related to its antioxidant and anti-inflammatory properties [6,7,8,9,10,11]. However, rutin is unstable and is easily converted into quercetin by rutin 3-glucosidase, which is present in TB grains during thermal processing [12,13]. This conversion leads to a bitter taste, which limits the diverse applications of TB in commercially processed foods, although quercetin has similar antioxidant activity with rutin [14,15].

Most studies have focused on the bioactivity of total TB extracts, but not TB flour extracts (TBFEs) [16,17]. It is noteworthy that hull and bran are not edible TB parts, and flour fractions are generally used in daily diets and processed foods. However, TBFE has considerably lower levels of rutin and quercetin than hull or bran fraction extracts [5]. Our previous study reported the enhancement of rutin content in TBFEs through hydrothermal treatments (autoclaving, boiling, or steaming). In addition, we also reported elevated antioxidant activity and intestinal absorption of TBFEs in a cultured cell line and in an ex vivo small intestinal sac model, respectively [18]. It was found that antioxidant activity was highly associated with rutin content [18]. There was also a significant enhancement in antioxidant activity, cellular uptake, and ex vivo intestinal transport efficiency of hydrothermally treated TBFEs compared with native TBFE or standard rutin [18].

To the best of our knowledge, little information is available regarding the bioavailability of bioactive compounds in TBFE and its effects on animal models. It is challenging to determine the bioavailability of the glycoside forms of quercetin (rutin or quercitrin) owing to their rapid metabolism through oral absorption and systemic circulation [19,20]. It was reported that they are hydrolyzed to their aglycone forms in the small or large intestine by intestinal bacterial glycosidase, resulting in several types of metabolites, such as glucuronides, sulfate conjugates, and methylated compounds [21,22]. A few studies were carried out to determine the in vivo effects of TB extracts on hyperlipidemia, obesity, hypertension, and inflammatory liver injury in rats [23,24,25,26]. Recently, the pharmacokinetics and associated metabolites of TB extracts were evaluated in beagle dogs following a single-dose co-administration with ethanol; however, the in vivo functional activity in alcoholic liver disease (ALD) was not demonstrated [27]. Indeed, TB extracts are promising agents for the treatment of ALD owing to its antioxidant and anti-inflammatory activities, which can be attributed to their high total polyphenol and flavonoid contents.

In this study, the pharmacokinetic profiles of native TBFE, three differently hydrothermal treated (autoclaving, boiling, or steaming) TBFEs, and standard rutin were evaluated after a single-dose oral administration in rats. Furthermore, the protective effect of TBFEs against ethanol-induced liver injury in rats was investigated by measuring the antioxidant enzyme activity and endogenous antioxidant level, lipid peroxidation, liver injury biomarkers, and calcium permeability after their repetitive oral administration for 28 days prior to ethanol administration.

## 2. Materials and Methods

### 2.1. Materials and Chemicals

TB grains were provided by Bongpyung-nongwon Co. Ltd. (Gangwon-do, Korea). Ethanol, tannic acid, acetic acid, l-ascorbic acid, sodium chloride, sodium acetate, acetonitrile, methanol, and sodium phosphate were acquired from Samchun Pure Chemical Co., Ltd. (Pyeongtaek, Korea). Rutin trihydrate was provided by Alfa Aesar (Ward Hill, MA, USA). Sulfatase from Helix pomatia, β-glucuronidase from bovine liver, quercetin, 4-(2-hydroxyethyl)-1-piperazineethanesulfonic acid (HEPES), ethylene glycol-bis (β-aminoethyl ether)-*N,N,N’,N’*-tetraacetic acid (EGTA), mannitol, and sucrose were purchased from Sigma-Aldrich (St. Louis, MO, USA). Phosphate-buffered saline (PBS) was provided by Welgene Inc. (Gyeongsan, Korea). Bio-Rad protein assay dye reagent from Bio-Rad Laboratories, Inc. (Hercules, CA, USA), superoxide dismutase (SOD) assay kit from Cayman Chemical Company (Ann Arbor, MI, USA), (+)-catechin, catalase (CAT) assay kit, and glutathione reductase (GR) assay kit from ENZO Life Sciences (Farmingdale, NY, USA), lipid peroxidation (malondialdehyde; MDA) assay kit from abcam (Cambridge, UK), 5,5-dithio-bis-(2-nitrobenzoic acid) (DTNB) from Thermo Fisher Scientific, Inc. (Waltham, MA, USA), and l-cysteine hydrochloride monohydrate from Junsei Chemical Co., Ltd. (Kyoto, Japan) were used.

### 2.2. Preparation of Rutin-Enriched TBFEs

Rutin-enriched TB flours were obtained after three different hydrothermal treatments (autoclaving, boiling, or steaming) performed at Sejong University, as previously described [15]. Native and hydrothermally treated TB flours (10 g) were extracted in 200 mL of 70% ethanol and sonicated (Bransonic 5800, Branson Ultrasonics, Danbury, CT, USA) at 40 °C for 30 min as previously reported [13,18]. The supernatants were used for further study after centrifugation (14,000× *g*) for 20 min at 4 °C, followed by evaporation at 65 °C to volatilize the ethanol solution and to obtain a three-fold concentrated solution for cell and animal experiments.

### 2.3. Analysis of Total Polyphenol, Total Flavonoid, Rutin, and Quercetin Contents in TBFEs

Total polyphenol concentrations were measured according to the Folin-Ciocalteu method [28]. Briefly, 80 μL of TBFEs were mixed and incubated with 20 μL of Folin–Ciocalteu phenol reagent (50%) in dark conditions. After 5 min, 100 μL of 2% sodium carbonate were added, and the mixture was further incubated for 30 min. The absorbance at 750 nm was measured using a microplate reader (SpectraMax^®^ M3, Molecular Devices, Sunnyvale, CA, USA). The concentrations of total polyphenols were presented as tannic acid equivalents (TAE g/100 mL extracts).

Total flavonoid concentrations were determined according to the method used by Zhishen et al. [29]. In brief, 30 μL of sodium nitrite (5%) was incubated with 50 μL of TBFEs. After 5 min, 60 μL of aluminum chloride hexahydrate (2%) was added and the solutions were incubated for 6 min. The solutions were further incubated for 11 min after adding 100 μL of sodium hydroxide (1 N). All experiments were performed at room temperature in dark conditions. The absorbance at 510 nm was measured using a microplate reader (SpectraMax^®^ M3, Molecular Devices). Total flavonoid content were expressed as catechin equivalents (CE g/100 mL extracts).

The concentrations of rutin and quercetin were determined using high-performance liquid chromatography (HPLC; Agilent 1100 series, Agilent Technologies, Santa Clara, CA, USA) equipped with a variable wavelength detector. Supelcosil™ LC-18 column (250 mm × 4.6 mm i.d., 5 μm, Supelco Inc., Bellefonte, PA, USA) was used and the mobile phase was methanol:acetonitrile:water containing 2.5% acetic acid (1:2:7, v/v/v). The samples were analyzed following filtration using a syringe filter (Advantec, Techigi, Japan). The wavelength and flow rate were set at 350 nm and 1 mL/min, respectively. The injection volume of the samples was 20 μL.

### 2.4. Animals

Six-week-old female Sprague-Dawley (SD) rats weighing 140–150 g were purchased from Koatech Co. (Gyeonggi-do, Korea). The animals were maintained in plastic laboratory animal cages in a clean animal rack at a controlled temperature (20 ± 2 °C), with a controlled relative humidity (60 ± 10%) and a 12 h light/dark cycle. They were provided with commercial standard food and water ad libitum. The rats were acclimated to their cages and rack for 7 days before treatment. All animal experiments were performed in accordance with the guidelines issued by the Animal and Ethics Review Committee of Seoul Women’s University (SWU IACUC-2019A-13), Korea.

### 2.5. Pharmacokinetic Study

Single-dose TBFEs or standard rutin (10 mL/kg) were administered to six female rats per group (~200 g) by oral gavage. The doses administered were as follows: native sample, 2.95 mg/kg rutin and 0.85 mg/kg quercetin; autoclaved sample, 15.65 mg/kg rutin and 0.45 mg/kg quercetin; boiled sample, 15.45 mg/kg rutin and 0.15 mg/kg quercetin; steamed sample, 12.10 mg/kg rutin and 0.15 mg/kg quercetin; standard rutin, equivalent to rutin concentration in the autoclaved sample. The administered doses were different because the contents of rutin in the native, autoclaved, boiled, and steamed TBFEs were different (Table 1). Blood samples were collected through the rats’ tail veins at 0, 0.08, 0.25, 0.5, 1, 1.5, 2, 4, 6, and 8 h after administration, and centrifuged at 3000× *g* for 15 min to separate the plasma. A volume of 100 μL of the plasma was acidified with 50 μL of 1 M acetic acid containing 2 mg/mL of l-ascorbic acid to prevent the loss of flavonoids because flavonoids are unstable under alkaline conditions [22,30]. All samples were stored at −80 °C before analysis.

β-glucuronidase and sulfatase were used to hydrolyze rutin metabolites in the plasma to quercetin aglycone [22]. A volume of 100 μL of the plasma was treated with 50 μL of β-glucuronidase (500 units/mL in 0.2% NaCl) and 50 μL of sulfatase (1000 units/mL in 34.2 mM NaCl). The treated samples were vortexed and mixed with 100 μL of 200 mM sodium acetate buffer (pH = 5), and the mixtures were incubated for 1 h at 37 °C. Each mixture was vortexed after two volumes of acetonitrile were added. After centrifugation at 10,000× *g* for 5 min at 4 °C, the supernatants were concentrated using a nitrogen evaporator (MG-3100, Eyela, Tokyo, Japan). The concentrated sample was then resuspended in 100 μL of methanol and analyzed by HPLC as described in Section 2.3. The following pharmacokinetic parameters were assessed using the Kinetica software (version 4.4, Thermo Fisher Scientific, Waltham, MA, USA): maximum concentration (C_max_), time to maximum concentration (T_max_), area under the plasma concentration-time curve (AUC), half-time (T_1/2_), and mean residence time (MRT).

### 2.6. Animal Treatments

Forty-two female rats were divided into seven groups of six animals and administered via oral gavage for 28 days as follows: (1) the normal control group was given distilled water (10 mL/kg) twice at 5-h interval; (2) the ethanol control group received 40% ethanol (10 mL/kg) 5 h post-administration of distilled water (10 mL/kg); (3–6) the TBFE groups were administered native or hydrothermally-treated TBFEs (10 mL/kg), respectively, 5 h prior to administration of 40% ethanol (10 mL/kg); and (7) the standard rutin group was treated with a standard rutin solution (equivalent to the rutin amount in the autoclaved sample, 10 mL/kg), 5 h prior to administration of 40% ethanol (10 mL/kg). All animals were euthanized using CO_2_ on the 29th day.

### 2.7. Liver Homogenization

Liver samples were collected, washed with ice-cold PBS, and chopped using scissors. Then, 1 g of the chopped liver samples were resuspended in 5 mL of ice-cold HEPES buffer (pH = 7.2, containing 1 mM EGTA, 210 mM mannitol, and 70 mM sucrose) and homogenized using an ultrasonic processor (Sonics & Materials Inc., Newtown, CT, USA). After centrifugation (1500× *g*, 3 min) at 4 °C, the Bradford assay was used to determine protein concentrations in the supernatants. The supernatants were then used for biochemical analysis.

### 2.8. Biochemical Analysis

Blood samples were collected from the rat posterior vena cava for serum biochemical analysis, as previously described [31]. A biochemical analyzer (TBA-120FR, Toshiba, Otawara, Japan) was used to analyze aspartate transaminase (AST), alanine aminotransferase (ALT), alkaline phosphatase (ALP), analyze triglyceride (TG), total cholesterol (TC), and calcium levels.

Antioxidant activities of enzymes such as SOD, CAT, and GR, were determined using chemical CAT, GR, and SOD assay kits, respectively, according to the manufacturers’ protocols [32,33,34]. Enzymatic activity was presented as units (U/mg). The MDA assay kit was used to determine lipid peroxidation according to the manufacturer’s protocol [35]. MDA content was presented as nmol/mg. The DTNB based on the Ellman’s method was used to determine glutathione (GSH) concentration [36]. GSH concentration was then presented as nmol of −SH nM/mg.

### 2.9. Statistical Analysis

Results were expressed as means ± standard deviations. One-way analysis of variance with Tukey’s test was performed using the SAS Ver.9.4 (SAS Institute Inc., Cary, NC, USA) to determine statistical significance between the control and treated groups at *p* values of < 0.05.

## 3. Results

### 3.1. Characterization of Antioxidant Compounds in Rutin-Enriched TBFEs

**Figure 1:**
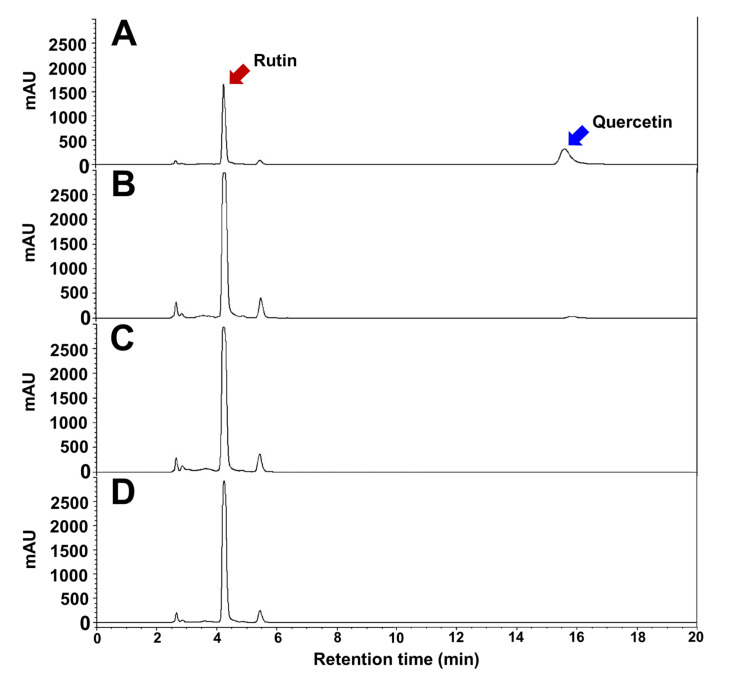
HPLC chromatograms of (**A**) native, (**B**) autoclaved, (**C**) boiled, and (**D**) steamed TBFEs.

Major bioactive compounds of TBFEs were evaluated by analyzing antioxidant components, such as rutin, quercetin, total polyphenols, and total flavonoids. HPLC chromatograms clearly show that rutin contents remarkably increased in all hydrothermally treated (autoclaved, boiled, and steamed) TBFEs compared with native TBFE (Figure 1). Quercetin level was more pronounced in native TBFE than in hydrothermally treated TBFEs. Table 1 demonstrates that rutin, total polyphenol, and total flavonoid contents increased in all hydrothermally treated TBFEs compared with those in native TBFE as previously described [18]. In particular, significantly high levels of rutin, total polyphenols, and total flavonoids were found in the autoclaved and boiled samples compared with those in the steamed one. This result indicates that rutin-enriched TBFEs can be obtained by hydrothermal treatment, which is efficient in preventing rutin conversion into quercetin, probably owing to the inactivation of rutin 3-glucosidase present TB grains.

### 3.2. Pharmacokinetics of Rutin-Enriched TBFEs

Plasma concentration-time profiles of native and rutin-enriched TBFEs in rats were investigated after a single-dose oral administration by measuring bioavailable flavonoid levels. Rutin, a glycoside of quercetin, is known to be hydrolyzed to its aglycon (quercetin) in the small or large intestine by the glycosidase present in intestinal bacteria following oral administration [21,22,37,38,39]. Quercetin is then metabolized to its glucuronides and sulfate conjugates (phase II metabolites) by oral absorption and systemic circulation [37,38,39]. Methylated metabolites of quercetin (isorhamnetin and tamarixetin) are reportedly present in plasma [21,22,40]. However, rutin, isorhamnetin or tamarixetin were not detected after oral administration of standard rutin (250 mg/kg) in our preliminary experiment. Zhao et al. also reported that there was no rutin, isorhamnetin, or tamarixetin in plasma, although orally administered concentrations of TB were higher than those used in this study [22]. Recently, Liu et al. demonstrated the pharmacokinetics and metabolites of TB extracts in the plasma of beagle dogs after hydrolysis with β-glucuronidase and sulfatase [27]. Hence, total quercetin in the plasma was analyzed as the main bioavailable flavonoid after hydrolysis with these two enzymes.

**Figure 2:**
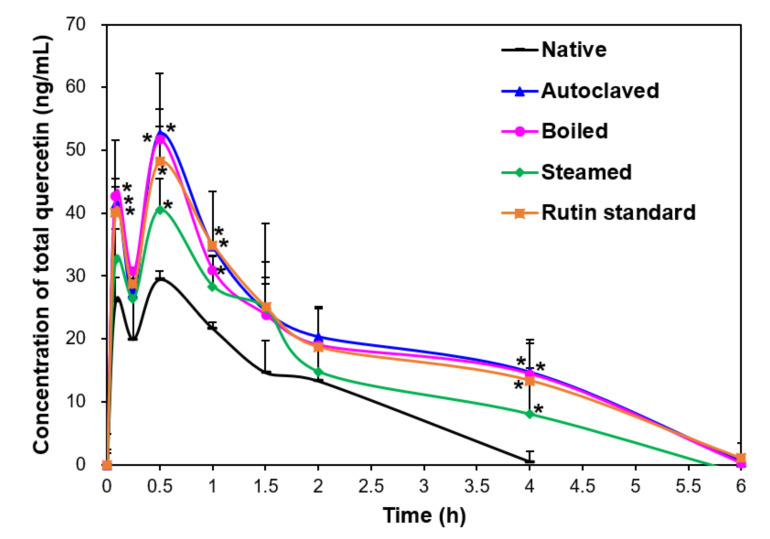
Plasma concentration-time curves of total quercetin after a single-dose oral administration of native, rutin-enriched TB flour extracts (TBFEs), and standard rutin in rats (*n* = 6). Asterisk (*) indicates significant differences from native TBFE at each time point (*p* < 0.05).

Figure 2 shows that rutin-enriched TBFEs had higher plasma total quercetin levels than native sample. Slightly lower total quercetin concentration was found in the steam sample, which is related to relatively low rutin and quercetin levels in this sample compared with those in the autoclaved and boiled samples (Table 1). Similar plasma total quercetin concentration compared with autoclaved and boiled TBFEs was found when standard rutin was administered, which is attributed to the same administered dose of standard rutin to the concentration measured in the autoclaved TBFE.

When pharmacokinetic parameters were calculated, C_max_ and AUC values were high in rutin-enriched TBFEs and standard rutin compared with those in native TBFE, implying their enhanced absorption (Table 2). On the other hand, slightly but statistically high T_1/2_ and MRT values of rutin-enhanced TBFEs were found compared with those of the native sample. These results suggest that hydrothermal treatment increases the half-life and residence time of total quercetin in the bloodstream. Oral absorption (0.04–0.05%) of rutin-enhanced TBFEs, calculated using AUC values, significantly increased compared with that of the native sample (0.02%). The pharmacokinetic parameters of standard rutin were similar to those of autoclaved and boiled TBFEs, which were related to the equivalent rutin amounts. Similar pharmacokinetic profiles were reported by Zhao et al. [22], but the low plasma total quercetin levels in the present study seem to be associated with lower rutin concentrations in TBFEs than those used in the study by Zhao et al. [22]. The fact that only quercetin was detected by hydrolysis of rutin metabolites with β-glucuronidase and sulfatase suggests that major bioavailable forms of orally administered rutin are quercetin glucuronide and quercetin sulfate. These results also suggest that orally administered rutin was rapidly metabolized during intestinal transit. Quercetin glucuronide and quercetin sulfate were detected at a maximum concentration in the plasma at 0.6–0.8 h post-oral ingestion of fried onions in humans, which contain quercetin as a major bioactive component [21], and is consistent with our findings.

### 3.3. Antioxidant Effects of TBFEs on Alcohol-Induced Oxidative Stress in Rat Livers

**Figure 3:**
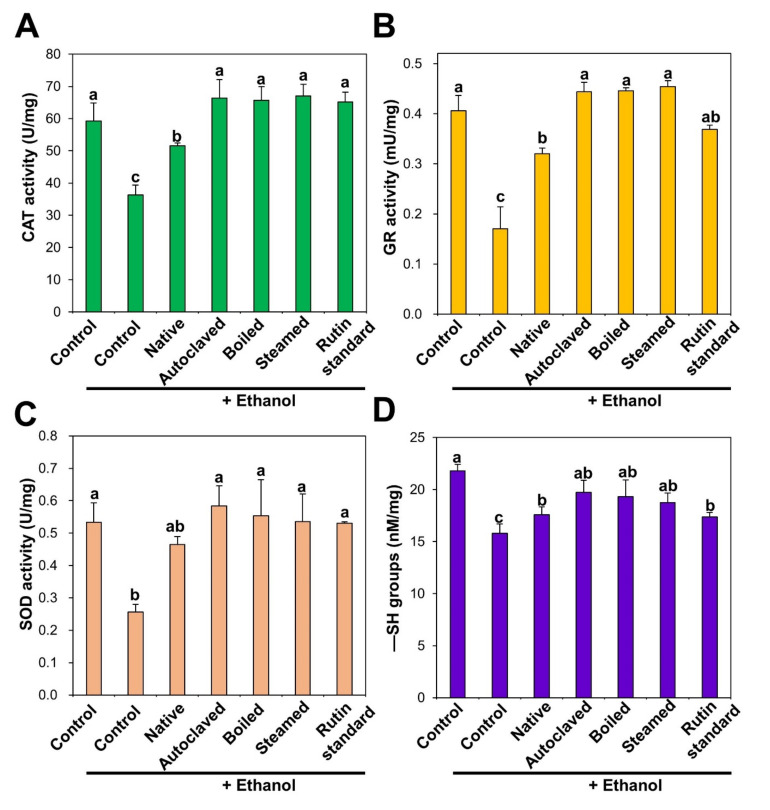
(**A**) catalase (CAT), (**B**) glutathione reductase (GR), (**C**) superoxide dismutase (SOD) activities, and (**D**) −SH groups level in the liver after 28-day repeated oral administration of native, rutin-enriched TBFEs, and standard rutin, 5 h prior to ethanol administration. Different lowercase letters (a, b and c) indicate significant differences among native, rutin-enriched (autoclaved, boiled, and steamed) TBFEs, and standard rutin (*p* < 0.05).

The functions of the antioxidant defense system were evaluated by measuring the levels of antioxidant activity of enzymes such as CAT, GR, and SOD, and an endogenous antioxidant, GSH, in rat livers after ethanol administration with or without pre-treatment with TBFEs for 28 days. The ethanol-induced ALD model was chosen because chronic ethanol ingestion increases the generation of reactive oxygen species, reduces intracellular antioxidant levels, and decreases antioxidant enzyme activities [41,42]. This consequently induces oxidative stress, especially in the liver, where ethanol metabolism mainly occurs [41,43]. CAT decomposes hydrogen peroxide into water and oxygen, and GR catalyzes the reduction of glutathione disulfide (GSSG) to GSH in the presence of nicotinamide adenine dinucleotide phosphate hydrogen (NADPH). SOD decomposes superoxide anion radicals into oxygen and hydrogen peroxide. The activities of all antioxidant enzymes in the liver were remarkably decreased by repetitive ethanol administration, whereas it was increased by native TBFE. Moreover, it was significantly increased and approached normal control levels following administration of rutin-enriched TBFEs and standard rutin (Figure 3A–C). It was reported that rutin treatment increased antioxidant activity against ethanol-related oxidative stress in the testis and liver of rats [44,45]. Protective effects of rutin against liver injury induced by biliary obstruction and type 2 diabetes were also demonstrated in animal models [46,47]. Recently, Sadauskiene et al. also demonstrated that the administration of buckwheat flower and leaf extracts had a significant effect on the antioxidant enzyme activity in the brain and liver of mice [48]. Furthermore, the serum antioxidant activity of buckwheat honey was reported in healthy humans [49].

Accordingly, decreased GSH concentration in the liver by ethanol administration was significantly increased by all TBFEs and standard rutin (Figure 3D). In particular, GSH level in the rat liver treated with rutin-enriched TBFEs was statistically similar to that found in the non-treated control group. GSH is an intracellular antioxidant that plays a role in scavenging free radicals [50]. Shenbagam et al. reported that rutin treatment could recover GSH content toward non-treated control level in alcohol-induced stress in the liver [45]. Yang et al. also demonstrated that the administration of TB extracts restored GSH levels that were lowered by alcohol-induced oxidative stress in the liver [51]. These results suggest that TBFEs have a protective role against ethanol-induced oxidative stress, which is attributed to high rutin, total polyphenol, and total flavonoid levels (Table 1).

### 3.4. Protective Effects of TBFEs against Alcohol-Induced Liver Injury in Rats

After repeated oral administration of ethanol for 28 days in rats, body weight gain, liver weight, relative liver weight, food intake, and water consumption were evaluated. Table 3 shows that rats that were administered ethanol had a significantly lower body weight gain and food intake than the non-treated control and TBFEs-treated groups. Relative liver weight was increased in rats that were administered ethanol, which is often observed owing to liver damage [52,53]. However, significant decrease in body weight or food intake and increase in relative liver weight were not observed in rats treated with TBFEs or standard rutin, indicating the potential role of TBFEs and rutin in protecting against ethanol-induced liver toxicity. Similar findings were reported, showing that rutin treatment can approximate body weight to normal following decreased body weight due to alcohol consumption [45]. Yang et al. also demonstrated that increased relative liver weight due to alcohol treatment was decreased when TB extracts were orally administered [51]. Recently, the protective activity of TB extract on alcohol-induced liver injuries was reported, which was related to its inhibitory effects on oxidative stress and mitochondrial cell death pathway [51].

**Figure 4:**
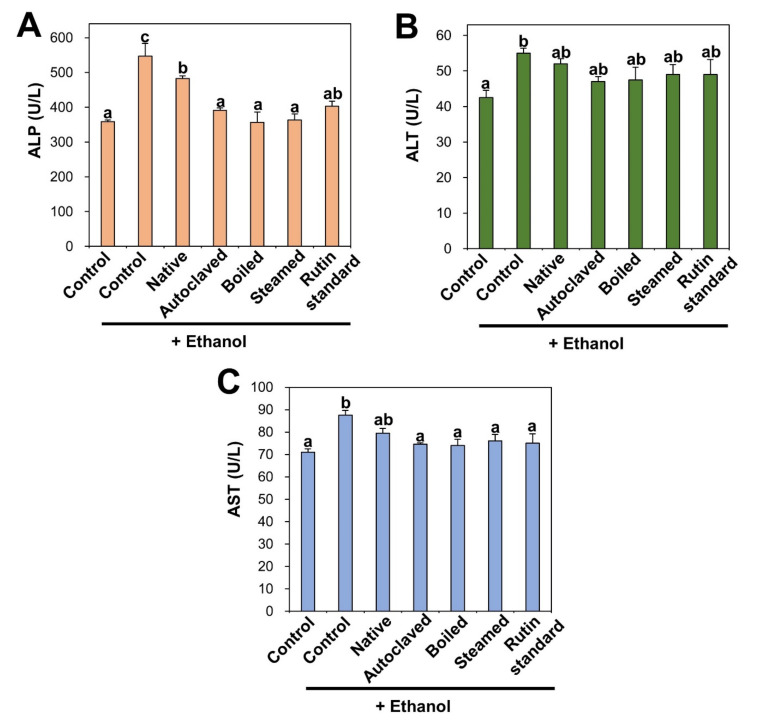
(**A**) alkaline phosphatase (ALP) (**B**) alanine aminotransferase (ALT) and (**C**) aspartate transaminase (AST) in serum after 28-day repeated oral administration of native, rutin-enriched TBFEs, and standard rutin, 5 h prior to ethanol administration. Different lowercase letters (a, b and c) indicate significant differences among native, rutin-enriched (autoclaved, boiled, and steamed) TBFEs, and standard rutin (*p* < 0.05).

Biochemical markers of liver injury such as ALP, ALT, and AST increased in rat serum after ethanol administration and these are indicators of ethanol-induced liver damage (Figure 4). On the other hand, the liver injury markers were slightly reduced by native TBFE treatment prior to ethanol administration, and more significantly reduced by rutin-enriched TBFEs and standard rutin. ALP, ALT, and AST are present in hepatocytes and released into the extracellular medium upon liver injury. It was reported that free radicals produced during ethanol metabolism in the liver can initiate the release of these enzymes [43,45,52,54]. These results clearly indicate that TBFEs have protective effects against alcohol-induced liver damage, probably related to their antioxidant activity (Figure 3). This is consistent with previous findings, showing decreased plasma ALT and AST levels following the oral intake of TB extract in rats that were administered alcohol [51].

**Figure 5:**
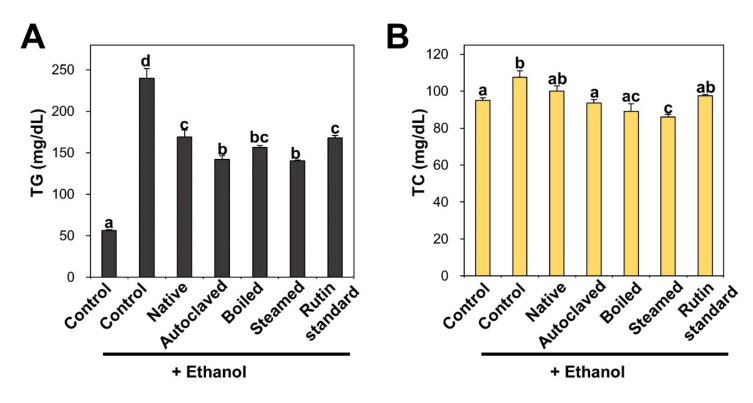
(**A**) triglyceride (TG) and (**B**) total cholesterol (TC) in serum after 28-day repeated oral administration of native, rutin-enriched TBFEs, and standard rutin, 5 h prior to ethanol administration. Different lowercase letters (a, b, c, and d) indicate significant differences among native, rutin-enriched (autoclaved, boiled, and steamed) TBFEs, and standard rutin (*p* < 0.05).

TG and TC accumulate in the liver owing to excessive nicotinamide adenine dinucleotide hydrogen (NADH) and acetyl-CoA produced from ethanol catabolism as a consequence of chronic alcohol abuse [42,55,56]. Figure 5 shows that rats that were repetitively administered ethanol had remarkably increased TG and TC serum levels compared with non-treated controls. Consistent with this, TBFE treatments prior to ethanol administration significantly reduced TG and TC concentrations. In particular, rutin-enriched TBFEs exerted a greater protective effect against TG and TC increase caused by ethanol ingestion than standard rutin treatment. The elevated levels of TG and TC in ALD models were reportedly decreased by quercetin administration [42], which is consistent with our findings. Reduced serum cholesterol level was also demonstrated by eating buckwheat cookies in clinical trials [57].

**Figure 6:**
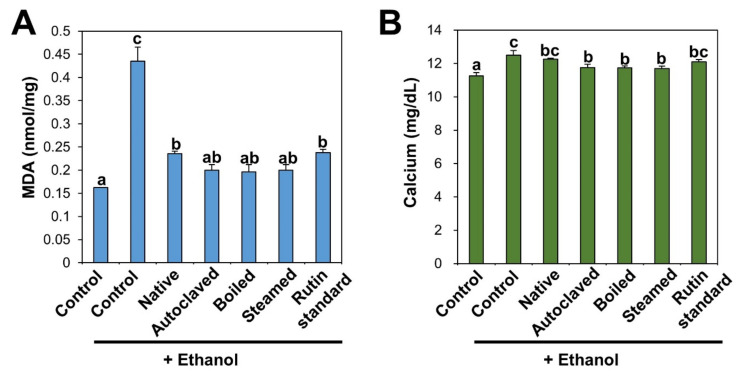
(**A**) MDA and (**B**) calcium in serum after 28-day repeated oral administration of native, rutin-enriched TBFEs, and standard rutin, 5 h prior to ethanol administration. Different lowercase letters (a, b and c) indicate significant differences among native, rutin-enriched (autoclaved, boiled, and steamed) TBFEs, and standard rutin (*p* < 0.05).

Induction of lipid peroxidation was determined by measuring MDA, a lipid peroxidation marker. MDA is one of the end-products of polyunsaturated fatty acid peroxidation and also used as a marker of oxidative stress [58]. Lipid peroxidation occurs through the oxidation of polyunsaturated fatty acids in the cell membrane by free radicals, leading to tissue damage [45,55,59]. Figure 6A shows that increased MDA level in ethanol-treated liver was significantly decreased by TBFE treatments. Slightly high antioxidant effects of rutin-enriched TBFEs on MDA formation were found compared with native sample and standard rutin. Previous findings demonstrated that the administration of rutin and TB extracts alleviated MDA levels in alcohol and cadmium-induced testicular injury and alcohol-induced liver injury, respectively [44,51].

Serum biochemical analysis revealed that calcium content was also remarkably increased following repetitive ethanol administration, but it was decreased by TBFE treatment, especially by rutin-enriched TBFEs compared with native TBFE and standard rutin (Figure 6B). TBFEs are composed of various components, such as proteins, vitamins, minerals, dietary fibers, rutin, quercetin, and other flavonoids, which contribute to an increase in their protective activity in the ALD model compared with standard rutin. It is known that free radicals generated during ethanol metabolism induce lipid peroxidation of the hepatocyte cell membrane, which increases calcium permeability in hepatocytes, thereby disrupting calcium homeostasis [54,60,61].

## 4. Conclusions

Rutin-enriched TBFEs were obtained using hydrothermal treatments (autoclaving, boiling, or steaming) and their pharmacokinetic profiles were assessed after a single-dose oral administration in rats. Moreover, the antioxidant activity and protective effects of TBFEs against ethanol-induced liver injury were evaluated following oral administration for 28 days in rats. The results demonstrated that rutin-enriched TBFEs had higher oral absorption and longer half-life than the native sample. Quercetin glucuronide and quercetin sulfate were the major bioavailable forms of TBFEs. In an ALD rat model, antioxidant enzyme activities and intracellular antioxidant levels were not affected by TBFE treatments, which contributes to their protective effect on liver injury. It was found that TBFEs had slightly higher antioxidant and protective effects on ethanol-induced liver damage than standard rutin. It is noteworthy that total daily intake of buckwheat is estimated to be about 32.44 mg/kg/day in Korea [62], which is much lower than the doses used in the present study. Nevertheless, these findings suggest the potential of TBFEs as antioxidant functional foods. Further study is required to elucidate the mechanism of action of TBFEs and their clinical effects on human.

## Figures and Tables

**Table 1:** The contents of rutin, quercetin, total polyphenols, and total flavonoids in native and rutin-enriched TBFEs. Different lowercase letters (a, b, c, and d) indicate significant differences among native and rutin-enriched (autoclaved, boiled, and steamed) TBFEs (*p* < 0.05).

Contents	Native	Autoclaved	Boiled	Steamed
Rutin (%) ^1^	0.57 ± 0.00 ^a^	3.03 ± 0.03 ^c^	2.97 ± 0.04 ^c^	2.50 ± 0.34 ^b^
Quercetin (%) ^1^	0.22 ± 0.00 ^a^	0.07 ± 0.00 ^b^	0.03 ± 0.00 ^d^	0.03 ± 0.00 ^c^
Total polyphenol (TAE g/100 mL extracts)	0.07 ± 0.00 ^a^	0.20 ± 0.00 ^c^	0.19 ± 0.00 ^c^	0.13 ± 0.00 ^b^
Total flavonoid (CE g/100 mL extracts)	0.03 ± 0.00 ^a^	0.17 ± 0.00 ^c^	0.17 ± 0.00 ^c^	0.09 ± 0.00 ^b^

^1^ Rutin and quercetin contents are expressed as g/100 g TB flours.

**Table 2:** Pharmacokinetic parameters and oral absorption of native, rutin-enriched TBFEs, and standard rutin after single-dose oral administrations in rats. Different lowercase letters (a, b, and c) indicate significant differences among native, rutin-enriched (autoclaved, boiled, and steamed) TBFEs, and standard rutin (*p* < 0.05).

Pharmacokinetic Parameters	Native	Autoclaved	Boiled	Steamed	Standard Rutin
T_max_ (h)	0.5	0.5	0.5	0.5	0.5
C_max_ (ng/mL)	26.4 ± 1.4 ^a^	52.7 ± 9.5 ^b^	51.7 ± 3.5 ^b^	40.5 ± 6.3 ^ab^	46.8 ± 2.3 ^b^
AUC (h × ng/mL)	46.6 ± 3.4 ^a^	110.4 ± 6.0 ^c^	107.8 ± 4.0 ^c^	75.5 ± 3.4 ^b^	103.4 ± 8.0 ^c^
T_1/2_ (h)	0.6 ± 0.1 ^a^	1.3 ± 0.1 ^b^	1.2 ± 0.0 ^b^	0.8 ± 0.1 ^a^	1.1 ± 0.2 ^b^
MRT (h)	1.2 ± 0.0 ^a^	2.1 ± 0.1 ^c^	2.0 ± 0.1 ^c^	1.6 ± 0.1 ^b^	1.8 ± 0.1 ^b^
Absorption (%)	0.02 ± 0.00 ^a^	0.05 ± 0.00 ^b^	0.05 ± 0.00 ^b^	0.04 ± 0.00 ^b^	0.04 ± 0.00 ^b^

**Table 3:** Body weight, liver weight, food intake, and water consumption in alcoholic liver disease (ALD) rat model after 28-day repeated oral administration of native, rutin-enriched TBFEs, and standard rutin, 5 h prior to ethanol administration. Different lowercase letters (a and b) indicate significant differences among native, rutin-enriched (autoclaved, boiled, and steamed) TBFEs, and standard rutin (*p* < 0.05).

Groups		+ Ethanol					
	Control	Control	Native	Autoclaved	Boiled	Steamed	Standard Rutin
Initial body weight (g)	175.9 ± 8.1 ^a^	175.1 ± 6.1 ^a^	174.5 ± 6.8 ^a^	175.7 ± 7.9 ^a^	177.2 ± 6.3 ^a^	173.9 ± 8.0 ^a^	175.6 ± 8.8 ^a^
Final body weight (g)	240.4 ± 4.8 ^a^	205.8 ± 6.6 ^b^	231.9 ± 6.1 ^a^	233.7 ± 11.7 ^a^	231.2 ± 2.1 ^a^	233.4 ± 5.7 ^a^	231.9 ± 9.9 ^a^
Body weight gain (g)	71.8 ± 10.1 ^a^	26.5 ± 9.6 ^b^	59.4 ± 6.1 ^a^	54.8 ± 8.3 ^a^	50.1 ± 1.0 ^a^	51.6 ± 3.5 ^a^	54.0 ± 11.6 ^a^
Liver weight (g)	7.8 ± 1.0 ^a^	8.7 ± 0.8 ^a^	8.5 ± 0.6 ^a^	8.7 ± 0.8 ^a^	8.5 ± 0.5 ^a^	8.2 ± 0.7 ^a^	8.5 ± 0.3 ^a^
Relative liver weight ^1^	3.5 ± 0.4 ^a^	4.2 ± 0.3 ^b^	3.8 ± 0.2 ^ab^	3.9 ± 0.4 ^ab^	3.8 ± 0.2 ^ab^	3.9 ± 0.3 ^ab^	3.9 ± 0.3 ^ab^
Food intake (g/day)	15.1 ± 1.3 ^a^	10.5 ± 1.0 ^b^	12.8 ± 1.4 ^ab^	12.3 ± 1.5 ^ab^	12.0 ± 1.7 ^ab^	12.1 ± 1.5 ^ab^	13.7 ± 1.3 ^a^
Water consumption (mL/day)	25.4 ± 2.7	30.4 ± 3.7 ^a^	30.6 ± 5.2 ^a^	33.2 ± 5.6 ^a^	27.1 ± 4.2 ^a^	28.8 ± 5.2 ^a^	29.7 ± 3.8 ^a^

^1^ Relative liver weight was calculated by liver g/100 g body weight.

## References

[1] Kang H.W. (2014). Antioxidant and anti-inflammation effects of water extract from buckwheat. Culi. Sci. Hos. Res..

[2] Xiao Y., Liu H., Wei T., Shen J., Wang M. (2017). Differences in physicochemical properties and in vitro digestibility between tartary buckwheat flour and starch modified by heat-moisture treatment. LWT-Food Sci. Technol..

[3] Zhu F. (2016). Chemical composition and health effects of tartary buckwheat. Food Chem..

[4] Lee L.-S., Choi E.-J., Kim C.-H., Sung J.-M., Kim Y.-B., Seo D.-H., Choi H.-W., Choi Y.-S., Kum J.-S., Park J.-D. (2016). Contribution of flavonoids to the antioxidant properties of common and tartary buckwheat. J. Cereal Sci..

[5] Yu J., Hwang J.-S., Oh M.S., Lee S., Choi S.-J. (2018). Antioxidant activity of ethanol extracts from common and tartary buckwheat milling fractions. Korean J. Food Sci. Technol..

[6] Nakamura K., Naramoto K., Koyama M. (2013). Blood-pressure-lowering effect of fermented buckwheat sprouts in spontaneously hypertensive rats. J. Funct. Foods.

[7] Sohn H.-Y., Kwon C.-S., Son K.-H., Kwon G.-S., Ryu H.-Y., Kum E.-J. (2006). Antithrombin and thrombosis prevention activity of buckwheat seed, *Fagopyrum esculentum* Moench. J. Korean Soc. Food Sci. Nutr..

[8] Yang N., Li Y.M., Zhang K., Jiao R., Ma K.Y., Zhang R., Ren G., Chen Z.-Y. (2014). Hypocholesterolemic activity of buckwheat flour is mediated by increasing sterol excretion and down-regulation of intestinal NPC1L1 and ACAT2. J. Funct. Foods.

[9] Han Y. (2009). Rutin has therapeutic effect on septic arthritis caused by *Candida albicans*. Int. Immunopharmacol..

[10] Tsai H., Deng H., Tsai S., Hsu Y. (2012). Bioactivity comparison of extracts from various parts of common and tartary buckwheats: Evaluation of the antioxidant-and angiotensin-converting enzyme inhibitory activities. Chem. Cent. J..

[11] Karki R., Park C.-H., Kim D.-W. (2013). Extract of buckwheat sprouts scavenges oxidation and inhibits pro-inflammatory mediators in lipopolysaccharide-stimulated macrophages (RAW264.7). J. Integr. Med..

[12] Kreft I., Fabjan N., Yasumoto K. (2006). Rutin content in buckwheat (*Fagopyrum esculentum* Moench) food materials and products. Food Chem..

[13] Cho Y.J., Bae I.Y., Inglett G.E., Lee S. (2014). Utilization of tartary buckwheat bran as a source of rutin and its effect on the rheological and antioxidant properties of wheat-based products. Ind. Crop. Prod..

[14] Ishiguro K., Morishita T., Ashizawa J., Suzuki T., Noda T. (2016). Antioxidative activities in rutin rich noodles and cookies made with a trace rutinosidase variety of tartary buckwheat (*Fagopyrum tataricum* Gaertn.),‘Manten-Kirari’. Food Sci. Technol. Res..

[15] Oh M., Oh I., Jeong S., Lee S. (2019). Optical, rheological, thermal, and microstructural elucidation of rutin enrichment in tartary buckwheat flour by hydrothermal treatments. Food Chem..

[16] Němcová L., Zima J., Barek J., Janovská D. (2011). Determination of resveratrol in grains, hulls and leaves of common and tartary buckwheat by HPLC with electrochemical detection at carbon paste electrode. Food Chem..

[17] Li F., Zhang X., Zheng S., Lu K., Zhao G., Ming J. (2016). The composition, antioxidant and antiproliferative capacities of phenolic compounds extracted from tartary buckwheat bran [*Fagopyrum tartaricum* (L.) Gaerth]. J. Funct. Foods.

[18] Jin H.-R., Yu J., Choi S.-J. (2019). Hydrothermal treatment enhances antioxidant activity and intestinal absorption of rutin in tartary buckwheat flour extracts. Foods.

[19] Graefe E.U., Wittig J., Mueller S., Riethling A.-K., Uehleke B., Drewelow B., Pforte H., Jacobasch G., Derendorf H., Veit M. (2001). Pharmacokinetics and bioavailability of quercetin glycosides in humans. J. Clin. Pharm..

[20] Yang C.-Y., Hsiu S.-L., Wen K.-C., Lin S.-P., Tsai S.-Y., Hou Y.-C., Chao P.-D.L. (2005). Bioavailability and metabolic pharmacokinetics of rutin and quercetin in rats. J. Food Drug. Anal..

[21] Mullen W., Edwards C.A., Crozier A. (2006). Absorption, excretion and metabolite profiling of methyl-, glucuronyl-, glucosyl- and sulpho-conjugates of quercetin in human plasma and urine after ingestion of onions. Br. J. Nutr..

[22] Zhao G., Zou L., Wang Z., Hu H., Hu Y., Peng L. (2011). Pharmacokinetic profile of total quercetin after single oral dose of tartary buckwheat extracts in rats. J. Agric. Food Chem..

[23] Wang M., Liu J.-R., Gao J.-M., Parry J.W., Wei Y.-M. (2009). Antioxidant activity of tartary buckwheat bran extract and its effect on the lipid profile of hyperlipidemic rats. J. Agric. Food Chem..

[24] Peng L., Zhang Q., Zhang Y., Yao Z., Song P., Wei L., Zhao G., Yan Z. (2020). Effect of tartary buckwheat, rutin, and quercetin on lipid metabolism in rats during high dietary fat intake. Food Sci. Nutr..

[25] Hou Z., Hu Y., Yang X., Chen W. (2017). Antihypertensive effects of tartary buckwheat flavonoids by improvement of vascular insulin sensitivity in spontaneously hypertensive rats. Food Funct..

[26] Lee C.-C., Shen S.-R., Lai Y.-J., Wu S.-C. (2013). Rutin and quercetin, bioactive compounds from tartary buckwheat, prevent liver inflammatory injury. Food Funct..

[27] Liu Y., Gan J., Liu W., Zhang X., Xu J., Wu Y., Yang Y., Si L., Li G., Huang J. (2019). Pharmacokinetics and novel metabolite identification of tartary buckwheat extracts in beagle dogs following co-administration with ethanol. Pharmaceutics.

[28] Appel H.M., Govenor H.L., D’Ascenzo M., Siska E., Schultz J.C. (2001). Limitations of folin assays of foliar phenolics in ecological studies. J. Chem. Ecol..

[29] Zhishen J., Mengcheng T., Jianming W. (1999). The determination of flavonoid contents in mulberry and their scavenging effects on superoxide radicals. Food Chem..

[30] Manach C., Morand C., Demigné C., Texier O., Régérat F., Rémésy C. (1997). Bioavailability of rutin and quercetin in rats. FEBS Lett..

[31] Jo M.-R., Bae S.-H., Go M.-R., Kim H.-J., Hwang Y.-G., Choi S.-J. (2015). Toxicity and biokinetics of colloidal gold nanoparticles. Nanomaterials.

[32] Peskin A.V., Winterbourn C.C. (2000). A microtiter plate assay for superoxide dismutase using a water-soluble tetrazolium salt (WST-1). Clin. Chim. Acta.

[33] Aebi H. (1984). Catalase in vitro. Methods Enzym..

[34] Smith I.K., Vierheller T.L., Thorne C.A. (1988). Assay of glutathione reductase in crude tissue homogenates using 5, 5′-dithiobis (2-nitrobenzoic acid). Anal. Biochem..

[35] Ohkawa H., Ohishi N., Yagi K. (1979). Assay for lipid peroxides in animal tissues by thiobarbituric acid reaction. Anal. Biochem..

[36] Ellman G.L. (1959). Tissue sulfhydryl groups. Arch. Biochem. Biophys..

[37] van der Woude H., Boersma M.G., Vervoort J., Rietjens I.M. (2004). Identification of 14 quercetin phase II mono-and mixed conjugates and their formation by rat and human phase II in vitro model systems. Chem. Res. Toxicol..

[38] Moon J.-H., Tsushida T., Nakahara K., Terao J. (2001). Identification of quercetin 3-O-β-D-glucuronide as an antioxidative metabolite in rat plasma after oral administration of quercetin. Free Radic. Bio. Med..

[39] Morand C., Crespy V., Manach C., Besson C., Demigné C., Rémésy C. (1998). Plasma metabolites of quercetin and their antioxidant properties. Am. J. Physiol..

[40] Lan K., Jiang X., He J. (2007). Quantitative determination of isorhamnetin, quercetin and kaempferol in rat plasma by liquid chromatography with electrospray ionization tandem mass spectrometry and its application to the pharmacokinetic study of isorhamnetin. Rapid Commun. Mass Spectrom..

[41] Lieber C.S. (1994). Alcohol and the liver: 1994 update. Gastroenterology.

[42] Tang Y., Gao C., Xing M., Li Y., Zhu L., Wang D., Yang X., Liu L., Yao P. (2012). Quercetin prevents ethanol-induced dyslipidemia and mitochondrial oxidative damage. Food Chem. Toxicol..

[43] Dahiru D., Obidoa O. (2008). Evaluation of the antioxidant effects of *Ziziphus mauritiana* lam. leaf extracts against chronic ethanol-induced hepatotoxicity in rat liver. Afr. J. Tradit. Complementary.

[44] Abarikwu S.O., Olufemi P.D., Lawrence C.J., Wekere F.C., Ochulor A.C., Barikuma A.M. (2017). Rutin, an antioxidant flavonoid, induces glutathione and glutathione peroxidase activities to protect against ethanol effects in cadmium-induced oxidative stress in the testis of adult rats. Andrologia.

[45] Shenbagam M., Nalini N. (2011). Dose response effect of rutin a dietary antioxidant on alcohol-induced prooxidant and antioxidant imbalance*—*A histopathologic study. Fundam. Clin. Pharm..

[46] Pan P.-H., Lin S.-Y., Wang Y.-Y., Chen W.-Y., Chuang Y.-H., Wu C.-C., Chen C.-J. (2014). Protective effects of rutin on liver injury induced by biliary obstruction in rats. Free Radic. Biol. Med..

[47] Liang W., Zhang D., Kang J., Meng X., Yang J., Yang L., Xue N., Gao Q., Han S., Gou X. (2018). Protective effects of rutin on liver injury in type 2 diabetic db/db mice. Biomed. Pharm..

[48] Sadauskiene I., Liekis A., Bernotiene R., Sulinskiene J., Kasauskas A., Zekonis G. (2018). The effects of buckwheat leaf and flower extracts on antioxidant status in mouse organs. Oxid. Med. Cell. Longev..

[49] Gheldof N., Wang X.-H., Engeseth N.J. (2003). Buckwheat honey increases serum antioxidant capacity in humans. J. Agric. Food Chem..

[50] Masella R., Di Benedetto R., Varì R., Filesi C., Giovannini C. (2005). Novel mechanisms of natural antioxidant compounds in biological systems: Involvement of glutathione and glutathione-related enzymes. J. Nutr. Biochem..

[51] Yang Q., Luo C., Zhang X., Liu Y., Wang Z., Cacciamani P., Shi J., Cui Y., Wang C., Sinha B. (2020). Tartary buckwheat extract alleviates alcohol-induced acute and chronic liver injuries through the inhibition of oxidative stress and mitochondrial cell death pathway. Am. J. Transl. Res..

[52] Owumi S.E., Odunola O.A., Aliyu M. (2012). Co-administration of sodium arsenite and ethanol: Protection by aqueous extract of *Aframomum longiscapum* seeds. Pharm. Res..

[53] Bak M.J., Truong V.-L., Ko S.-Y., Nguyen X.N.G., Ingkasupart P., Jun M., Shin J.Y., Jeong W.-S. (2016). Antioxidant and hepatoprotective effects of procyanidins from wild grape (*Vitis amurensis*) seeds in ethanol-induced cells and rats. Int. J. Mol. Sci..

[54] Sebai H., Jabri M.-A., Souli A., Hosni K., Rtibi K., Tebourbi O., El-Benna J., Sakly M. (2015). Chemical composition, antioxidant properties and hepatoprotective effects of chamomile (*Matricaria recutita* L.) decoction extract against alcohol-induced oxidative stress in rat. Gen. Physiol. Biophys..

[55] El-Newary S.A., Shaffie N.M., Omer E.A. (2017). The protection of *Thymus vulgaris* leaves alcoholic extract against hepatotoxicity of alcohol in rats. Asian Pac. J. Trop. Med..

[56] Koch O.R., Fusco S., Ranieri S.C., Maulucci G., Palozza P., Larocca L.M., Cravero A.A., Farre S.M., De Spirito M., Galeotti T. (2008). Role of the life span determinant P66^shcA^ in ethanol-induced liver damage. Lab. Investig..

[57] Wieslander G., Fabjan N., Vogrinčič M., Kreft I., Janson C., Spetz- Nyström U., Vombergar B., Tagesson C., Leanderson P., Norbäck D. (2011). Eating buckwheat cookies is associated with the reduction in serum levels of myeloperoxidase and cholesterol: A double blind crossover study in day-care centre staffs. Tohoku J. Exp. Med..

[58] Ayala A., Muñoz M.F., Argüelles S. (2014). Lipid peroxidation: Production, metabolism, and signaling mechanisms of malondialdehyde and 4-hydroxy-2-nonenal. Oxid. Med. Cell. Longev..

[59] Bourogaa E., Nciri R., Mezghani-Jarraya R., Racaud-Sultan C., Damak M., El Feki A. (2013). Antioxidant activity and hepatoprotective potential of *Hammada scoparia* against ethanol-induced liver injury in rats. J. Physiol. Biochem..

[60] Xiao Z.-M., Li L.-J., Yu S.-Z., Lu Z.-N., Li C.-Y., Zheng J.-Q. (2005). Effects of extracellular Ca^2+^ influx and intracellular Ca^2+^ release on ethanol-induced cytoplasmic Ca^2+^ overload in cultured superior cervical ganglion neurons. Neurosci. Lett..

[61] Powell L.W. (1975). The role of alcoholism in hepatic iron storage disease. Ann. N. Y. Acad. Sci..

[62] Korea Health Industry Development Institute (KHIDI) National Food & Nutrition Statistics. https://www.khidi.or.kr/kps/dhraStat/result2?menuId=MENU01653&gubun=sex&year=2018.

